# Type I Collagen Aging Increases Expression and Activation of EGFR and Induces Resistance to Erlotinib in Lung Carcinoma in 3D Matrix Model

**DOI:** 10.3389/fonc.2020.01593

**Published:** 2020-09-10

**Authors:** Thomas Sarazin, Guillaume Collin, Emilie Buache, Laurence Van Gulick, Céline Charpentier, Christine Terryn, Hamid Morjani, Charles Saby

**Affiliations:** ^1^Université de Reims Champagne-Ardenne, Unité BioSpecT, EA7506, SFR CAP-Santé, UFR de Pharmacie, Reims, France; ^2^Université de Reims Champagne Ardenne, Plate-forme Imagerie Cellulaire et Tissulaire (PICT), Reims, France

**Keywords:** Type I collagen, aging, EGFR, Erlotinib, resistance

## Abstract

Type I collagen is the major structural component of lung stroma. Because of its long half-life, type I collagen undergoes post-translational modifications such as glycation during aging process. These modifications have been shown to impact the structural organization of type I collagen fibers. In the present work we evaluated the impact of collagen aging on lung carcinoma cells response to erlotinib-induced cytotoxicity and apoptosis, and on Epidermal Growth Factor Receptor (EGFR) expression and phosphorylation. To this end, experiments were performed in 2D and 3D matrix models established from type I collagen extracted from adult (10 weeks-old) and old (100 weeks-old) rat's tail tendons. Our results show that old collagen induces a significant increase in EGFR expression and phosphorylation when compared to adult collagen in 3D matrix but not in 2D coating. Such modification was associated to an increase in the IC_50_ of erlotinib in the presence of old collagen and a lower sensitivity to drug-induced apoptosis. These data suggest that collagen aging confers resistance to the cytotoxic and apoptotic effects of therapies targeting EGFR kinase function in lung carcinoma. Moreover, our data underline the importance of the 3D matrix environment in this process.

## Introduction

Lung cancer is one of the leading causes of cancer-related death in the world (1, 2). Among causes responsible for developing lung cancer, cigarette smoking is the most recognized risk factor (3). Importantly, risk of developing lung cancer increases considerably with age, with a peak of incidence between 50 and 65 years old. There are different lung cancer subtypes. The most frequent histologic subtype is the adenocarcinoma (4). This subtype is composed of 40% of non-small-cell lung carcinoma (NSCLC). The principal treatment for such cancer is surgery followed by radiotherapy, chemotherapy, and targeted therapies against EGFR kinase function.

EGFR is amplified in about 80% of patients with NSCLC (5). EGFR mutations can lead to constitutive activation of signaling pathways such as PI3K/Akt/mTOR, which are involved in cancer cell survival. EGFR mutations have been shown to activate constitutively anti-apoptotic signaling pathways, thus inducing a resistance to therapies targeting EGFR kinase domain such as erlotinib. Erlotinib has shown efficacy against mutated forms of the receptor that are constitutively active (exon 19 deletion and L858R mutation on exon 21). At the opposite, the mutation T790M, which can be acquired during erlotinib treatment is observed in 50% of patients presenting failure after treatment.

To understand the mechanisms of resistance to targeted therapies like erlotinib, it is important to decipher the relationship between cancer cells and their microenvironment. It is well-known that the extracellular matrix (ECM) plays a crucial role in the regulation of tumor progression (6). In fact, ECM modulates cell proliferation, cell migration, tumor invasion, and can also promote resistance to therapies (7). The major component of ECM in several organs is type I collagen. One particularity of type I collagen is that its structural organization plays an important role in the modulation of tumor behavior. It has been shown that modifications of collagen organization (8), but also degradation (9–11) and aging (12–14) can affect several cancer hallmarks.

During chronological aging, type I collagen undergoes non-enzymatic post-translational modifications such as glycation, that results in the formation of Advanced Glycation End-product (AGE) (15). These AGEs lead to an increase in collagen cross-links (16), which have an impact on structural organization of the matrix protein (17, 18). In fact, it has been shown that these cross-links increase type I collagen fibers straightness and rigidity, whereas it decreases fibers length and width. Our team have shown that collagen aging is able to induce an increase in cell proliferation in fibrosarcoma and epithelial-like breast carcinoma (13, 14). Such process was also able to protect epithelial-like breast carcinoma against collagen-induced apoptosis (10, 14).

More recently, Chang et al. have shown that an increase of type I collagen rigidity rendered NSCLC cells A549 more resistant to erlotinib (8). It is important to note that in this work, experiments have been carried out in 2D collagen coating. Here we propose to study the effect of age-related modifications of type I collagen on A549 and transformed bronchial BZR cells sensitivity to erlotinib, in a 2D coating and 3D matrix models. We demonstrated that collagen aging confers resistance to erlotinib only in 3D matrix models. Our data suggest that resistance acquisition is associated to an increase of EGFR expression and phosphorylation in A549 and BZR cells. Moreover, our data underline the importance of the 3D environment in this process.

## Materials and Methods

### Cell Culture

The human lung carcinoma cell lines A549 (CCL-185) and the bronchial transformed cell line BZR (CRL-9483) were purchased from the American Type Culture Collection (ATCC). Both cell lines were cultured in DMEM (4,5 g/l glucose) with Glutamax I (PAN-Biotech, p04-04500) supplemented with 10% fetal bovine serum (Dominique Dutscher, S1810-500) and 1% penicillin-streptomycin (Invitrogen, 15140). Cultures were maintained at 37°C in a humidified atmosphere containing 5% CO2 (v/v). Cells were routinely passaged at preconfluency using 0.05% trypsin, 0.53 mM EDTA (Invitrogen, 25300) and screened for the absence of mycoplasma using PCR methods.

### Preparation and Characterization of Type I Collagen

Fibrillar native type I collagen was extracted from tail tendons of 10 weeks old (adult) and 100 weeks old (old) rats and prepared as already described (19). The animal procedure was approved by the local ethics committee in animal experimentation of Reims Champagne-Ardenne (C2EA, registration 56, France) and the experiments were performed in accordance with European directive 2010/63/UE. Briefly, type I collagen was extracted from tail tendons of Wistar rats (Janvier) using 0.5M acetic acid at 4°C, in the presence of protease inhibitors. Then type I collagen was specifically precipitated with NaCl 0.7M, and centrifuged. The precipitate was then re-suspended in 18 mM acetic acid, and salts used during the precipitation step were eliminated by dialysis against distilled water for 1 week at 4°C. Finally, the collagen was characterized as described in our previous work, before use (13, 14).

### 2D and 3D Cell Culture

2D and 3D cell culture experiments were performed in 24-well plates. For 2D cell cultures, each well was coated with 5 μg/cm^2^ of adult or old collagens solubilized in 0.018 M acetic acid. Coated substrates were dried overnight at room temperature under sterile conditions and rinsed twice in cold PBS (Invitrogen) before cell plating. For 3D cell culture, cells were resuspended in 100 μl fetal bovine serum and mixed with a solution containing 100 μl of 10× culture medium DMEM (Gibco, 52100), 100 μl NaHCO_3_ (0.44 M), 100 μl H_2_O, 90 μl NaOH 0.1 M, 10 μl glutamine 200 mM, and 500 μl collagen 3 mg/mL. Then, 1 mL/well of this solution was deposited in 24-well-plates, and gels were polymerized at 37°C during 30 min. Finally, 1 mL of complete culture medium was added on top of each gels and the plates were incubated at 37°C. To count the cells at the end of the experiment, the covering medium was removed, and cell populated gels were digested with collagenase P (2 mg/mL—Roche, 11213873001). Viability and cellular density of this suspension were determined by phase contrast microscopy using Kova® Glasstic® Slides (Kova International Inc, 87144). In some experiments, cells were treated with EGFR pharmacological inhibitors erlotinib (Selleckchem, No.OSI-744), at 18 μM.

### Laser Scanning Microscopy

Images were acquired with a laser scanning microscope LSM 710 NLO (Carl ZEISS SAS, Marly le roi, France) coupled with CHAMELEON femtosecond Titanium-Sapphire Laser (Coherent, USA) and managed with ZEN Software (Carl ZEISS SAS, Marly le roi, France) with 40x (ON: 1) objective lens. Excitation wavelength of Calcein (Invitrogen, C3100) was 488 nm Argon ion laser line, and fluorescence emission was collected using 500–560 nm bandpass filter. Second Harmonic Generation (SHG) signal was collected with 420–440 nm bandpass filter with excitation from femtosecond laser at 860 nm.

### Image Analysis

Visualization of A549 cells cultured on 2D coating of type I collagen was performed using ImageJ software (U.S. National Institutes of Health, Bethesda, MD). For the visualization of A549 cells in 3D matrices of type I collagen, 3D image reconstruction was processed with Imaris software (Bitplane, UK), using Z stack from samples (Z step:0.5 μm).

### EGFR Inhibition

Cell viability assay was assessed in 24-well-plates. For 2D and 3D cell cultures, 1.5×10^4^ cells (A549) or 3 × 10^4^ cells (BZR) were seeded in each well, in culture medium containing 2% fetal bovine serum. After 24 h, culture medium was replaced with fresh medium containing erlotinib, to obtain final concentration of (0; 1; 2.5; 10; 25; 100 μM)/well. After 72 h, cells were harvested and counted using phase contrast microscopy, and IC_50_ was determined for each condition.

### Quantification of Apoptosis

Cells were cultured in type I collagen 3D matrices supplemented with 2% fetal bovine serum. After 36 h, 1 × 10^5^ cells were harvested for each condition using collagenase P at 2 mg/mL, washed twice with PBS and tested for three parameters: Annexin V positive cells using Muse® Annexin V and Dead Cell Assay Kit (Millipore, MCH100105), reactive oxygen species using Muse® Oxydative Stress Kit (Millipore, MCH100111) and caspase 3 and 7 activity using Muse® Caspase-3/7 Assay Kit (Millipore, MCH100108), all according to the manufacturer's instructions.

### Western Blotting

Cells were seeded at a density of 100 × 10^3^ cellules/mL in adult and old type I collagen 3D matrices, with 2% fetal bovine serum. After 96 h, cells were harvested using collagenase P at 2 mg/mL, washed twice with PBS, and lysed with RadioImmuno Precipitation Assay (RIPA) buffer (Thermo Fisher Scientific, 89900), supplemented with Halt™ Protease and Phosphatase Inhibitor Cocktail 1× (Thermo Scientific, 78442). Cell lysates were sonicated and clarified by centrifugation at 14,000× g at 4°C for 15 min. Then, total protein content was estimated by bicinchoninic acid (BCA) assay method (Thermo Scientific, 23227), and proteins were separated by SDS-PAGE and transferred to a nitrocellulose membrane. Membranes were blocked with Tris Buffered Saline (TBS) (0.02 M Tris-HCl, 0.137 M NaCl, pH 7.6) containing 0.1% Tween (TBS-T) and 5% Bovine Serum Albumin (BSA) at room temperature during 1 h and incubated overnight at 4°C with anti-EGFR (Cell signaling Technology, #2232) or anti-GAPDH antibodies (Cell signaling Technology, #5174). Membranes were washed with TBS-T and incubated with peroxidase conjugated anti-rabbit secondary antibody (Cell signaling Technology, #7074) at room temperature for 1 h. Chemiluminescent detection was performed by using an ECL Prime Kit (GE Healthcare, RPN2236). Electrophoretic images were analyzed with ImageJ software.

### EGFR Activation

Cells were serum deprived for 10 h, then harvested and seeded at a density of 100 × 10^3^ cellules/mL in 3D matrices of adult and old type I collagen, in presence of 2% of fetal bovine serum. After 24 h, culture medium was replaced with new medium containing 15 μM of erlotinib or DMSO for the control condition. After 72 h of treatment, cells were harvested and washed two time in DPBS. pEGFR/total EGFR ratio was determined using the Muse® EGFR-RTK activation dual detection kit Dead Cell Assay Kit **(**Millipore, MCH200102), according to the manufacturer's instructions.

### Statistical Analysis

Data are presented as mean ± standard error of the mean (SEM) from three independent experiments. Statistical significance was assessed with Student's *t*-test, or with one-way ANOVA, followed by Tukey's multiple comparison test. *p*-values < 0.05 were considered as significant (^*^p < 0.05; ^**^p < 0.01; ^***^p < 0.001).

## Results

### Collagen Aging Impacts Cell Morphology in 3D

**Figure 1:**
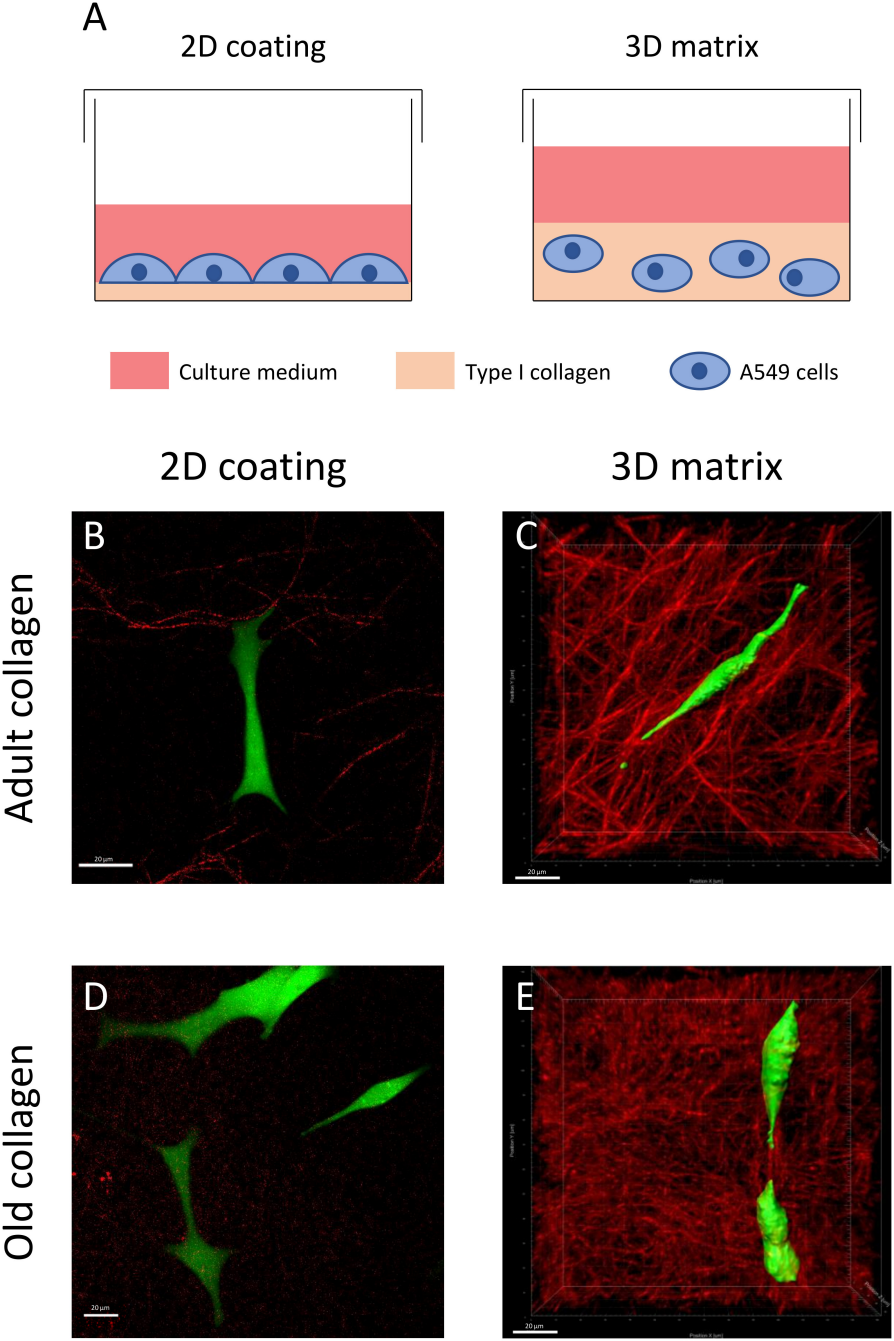
Effect of type I collagen aging on cell morphology in 2D vs. 3D models. **(A)** For 2D cultures, cells were plated on a thin coating (5 μg/cm^2^) of collagen. For 3D cultures, the cells were embedded into a collagen matrix (1.5 mg/mL final concentration). For confocal and SHG images, cells were seeded at a density of 5 × 10^3^ cells/mL either on 2D coating of adult **(B)** or old collagen **(D)**, or in 3D matrices of adult **(C)**, and old collagen **(E)**. After 24 h, cells were treated with 1 μM of calcein-AM for 1 h. For each condition, we show a representative confocal image of A549 cells (green) and an SHG image of collagen (red). Scale bars represent 20 μm.

As shown in Figure 1, we aimed to characterize morphology of A549 cells in adult and old type I collagen in 2D vs. 3D models. To this end, cells were plated on 2D coating or embedded in 3D type I collagen matrix for 24 h (Figure 1A). Type I collagen network was characterized by SHG, and images showed that adult type I collagen exhibited longer and thicker fibers than old collagen, both in 2D (Figures 1B,D) and 3D conditions (Figures 1C,E). Cell morphology was observed using confocal fluorescence microscopy. There were no significant changes in cell morphology between adult (Figure 1B) and old (Figure 1D) 2D type I collagen coating conditions. In fact, the cells exhibited in both conditions an epithelial morphology, with a large cytoplasm and several cytoplasmic protrusions. We then analyzed aged-related morphology changes in 3D matrix model. At the opposite of 2D collagen coating condition, cells cultured in 3D matrices exhibited an elongated form with less and longer protrusions (Figures 1C,E). Moreover, such prominent protrusions were aligned with the collagen fiber axis. Finally, when compared to 3D adult matrix (Figure 1C), A549 cells exhibited a less elongated form, but still with two prominent protrusions in 3D old matrix (Figure 1E).

### Collagen Aging Promotes Lung Cancer Cells Resistance to Erlotinib

**Figure 2:**
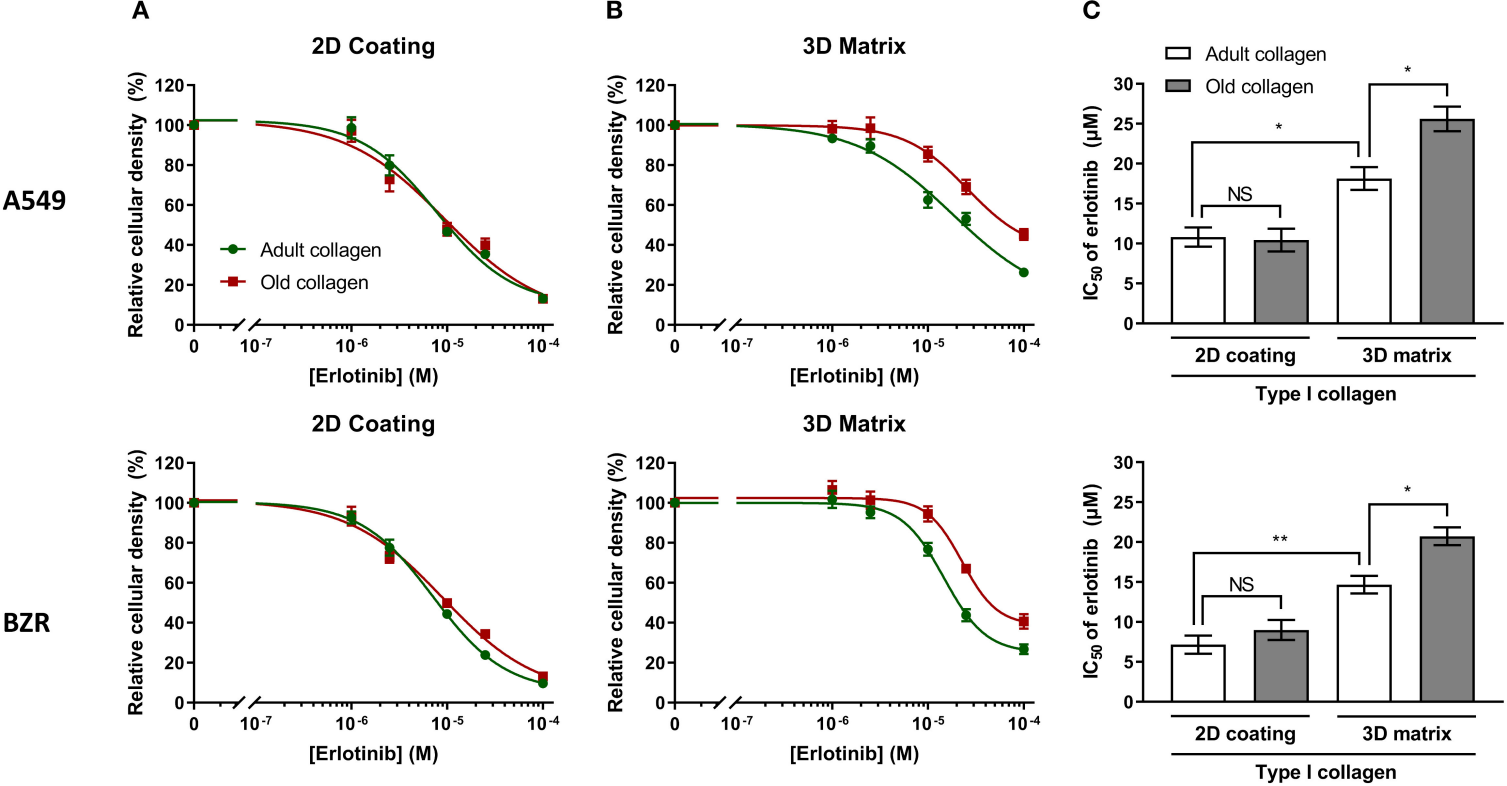
Effect of collagen aging on erlotinib IC_50_, in 2D vs. 3D models. A549 and BZR cell lines were seeded at a density of 1.5 × 10^4^ cells/mL and 3 × 10^4^ cells/mL, respectively. After 24 h, culture medium was replaced with fresh medium containing erlotinib, to obtain final concentration of (0; 1; 2.5; 10; 25; 100 μM)/well. After 72 h of culture with erlotinib, cells were harvested, and viable cell density was evaluated by phase contrast microscopy. The graphs represent erlotinib dose response curves of A549 cells (upper panel) and BZR cells (lower panel) cultured in adult and old collagen **(A)** in a 2D coating model or **(B)** in a 3D matrix model. **(C)** The histogram shows the IC_50_ of erlotinib for A549 cells (upper panel) and BZR cells (lower panel), cultured in adult vs. old collagen, in 2D vs. 3D model. Values represent the mean ± SEM (*p < 0.05, **p < 0.01, N.S = Not Significant).

Chang et al. have shown that in a 2D collagen coating model, an increase of type I collagen rigidity rendered NSCLC cells A549 more resistant to erlotinib (8). Previous studies from our group have shown that age-related modifications of type I collagen were linked to an increase of its rigidity (17, 18), and could modulate tumor cells behavior when used in a more physiological 3D model. Since lung carcinoma cells are confronted to a collagen rich microenvironment, we decided to investigate the effect of collagen aging on the toxicity of erlotinib on A549 and BZR cells by determining the IC_50_ of erlotinib in adult and old collagen, for both cell lines. To this end, cells were treated with different concentrations of erlotinib on 2D collagen coating, and in 3D matrix models. Then, cell viability was assessed by phase contrast microscopy. Figure 2A shows that in 2D, the sensitivity of A549 cells (upper panel) and BZR cells (lower panel) to erlotinib was similar in both collagens, with respective erlotinib IC_50_ of 10 and 8 μM (Figure 2C). In 3D matrix model, old collagen was able to protect A549 cells (upper panel) and BZR cells (lower panel) against erlotinib cytotoxicity (Figure 2B). In fact, erlotinib IC_50_ was 18 μM in adult collagen and 26 μM in old collagen for A549 cells, and 15 μM in adult collagen and 21 μM in old collagen for BZR cells (Figure 2C). We also evaluated cell growth for both cell lines in 2D and in 3D. A549 cells exhibit a higher cell growth rate in old collagen, when compared to the adult collagen in 3D but not in 2D, whereas BZR exhibit the same cell growth rate in both collagen and in both conditions (data not shown).

### Collagen Aging Reduces Erlotinib Induced Apoptosis

**Figure 3:**
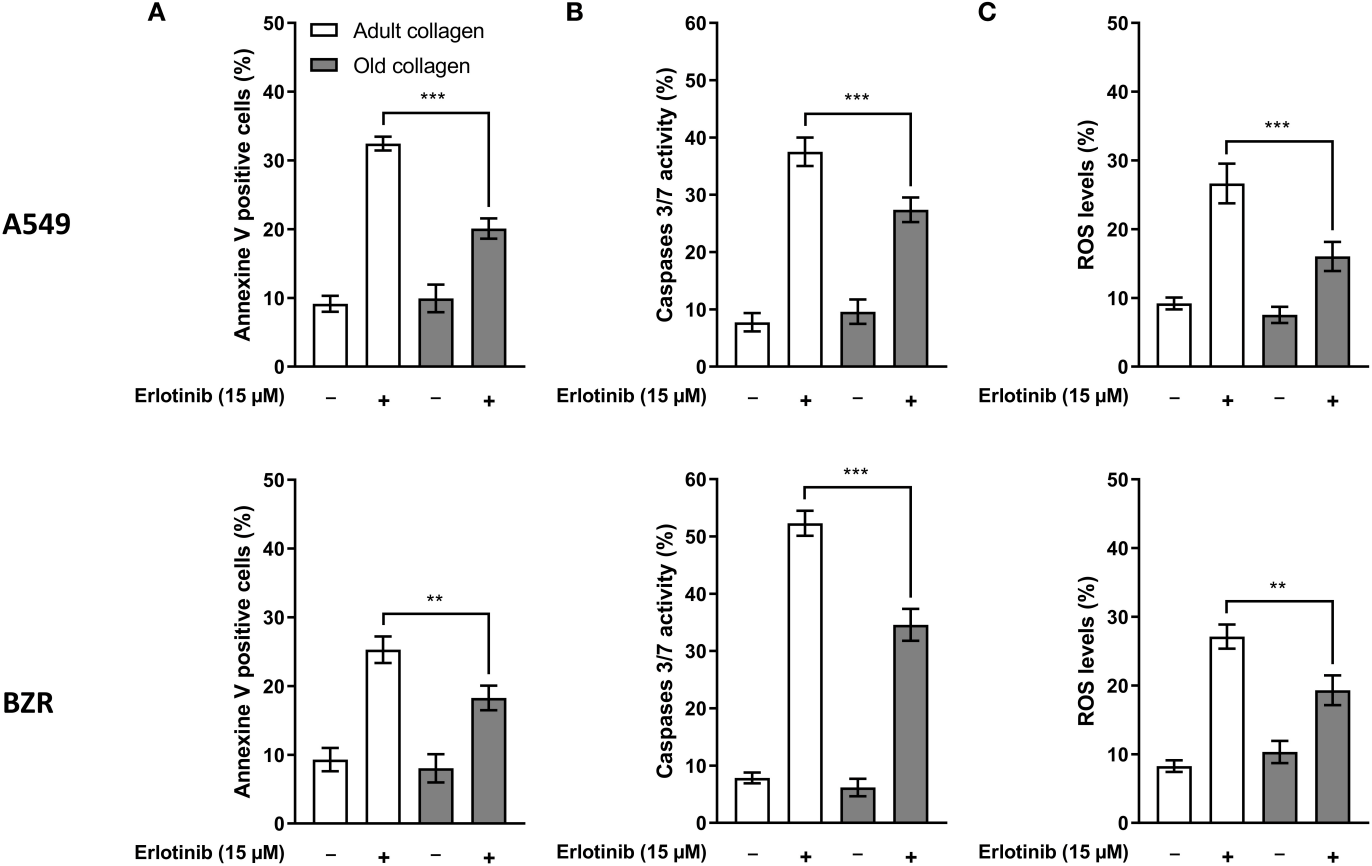
Effect of collagen aging on erlotinib induced apoptosis in a 3D collagen model. A549 cells (upper panel) and BZR cells (lower panel) were cultured in adult and old type I collagen 3D matrices, with or without 15 μM of erlotinib. After 36 h, cells were harvested and tested for three parameters. **(A)** Annexin V positive cells, **(B)** caspase 3 and 7 activity, and **(C)** reactive oxygen species. Values represent the mean ± SEM (**p < 0.01, ***p < 0.001).

A study from Shan et al. has shown that erlotinib was able to induce apoptosis in A549 cells (20). Here we decided to assess whether old collagen was able to decrease erlotinib-induced apoptosis in A549 and BZR cells. To this end, cells were cultured in 3D matrices of adult and old collagen in presence or not of erlotinib, and three apoptosis markers were quantified: (i) annexin V, (ii) caspases 3/7 activity, and (iii) the Reactive Oxygen Species level (ROS). As shown in Figure 3A, for both cell lines erlotinib induced an increase in the percentage of annexin V positive cells in both collagens, but the level observed in old collagen was significantly lower than that observed in adult collagen. In agreement with the annexin V data, Figure 3B shows that erlotinib induced an increase in caspase 3/7 activity in both collagens. The rate of caspase 3/7 positive cells was lower in old collagen when compared to adult collagen. Finally, Figure 3C shows that ROS level was increased in both collagens in the presence of erlotinib. However, the level observed in old collagen was lower than that observed in adult collagen. These data suggest that in a 3D matrix model, collagen aging protects A549, and BZR cells against erlotinib-induced apoptosis.

### Collagen Aging Promotes EGFR Expression

**Figure 4:**
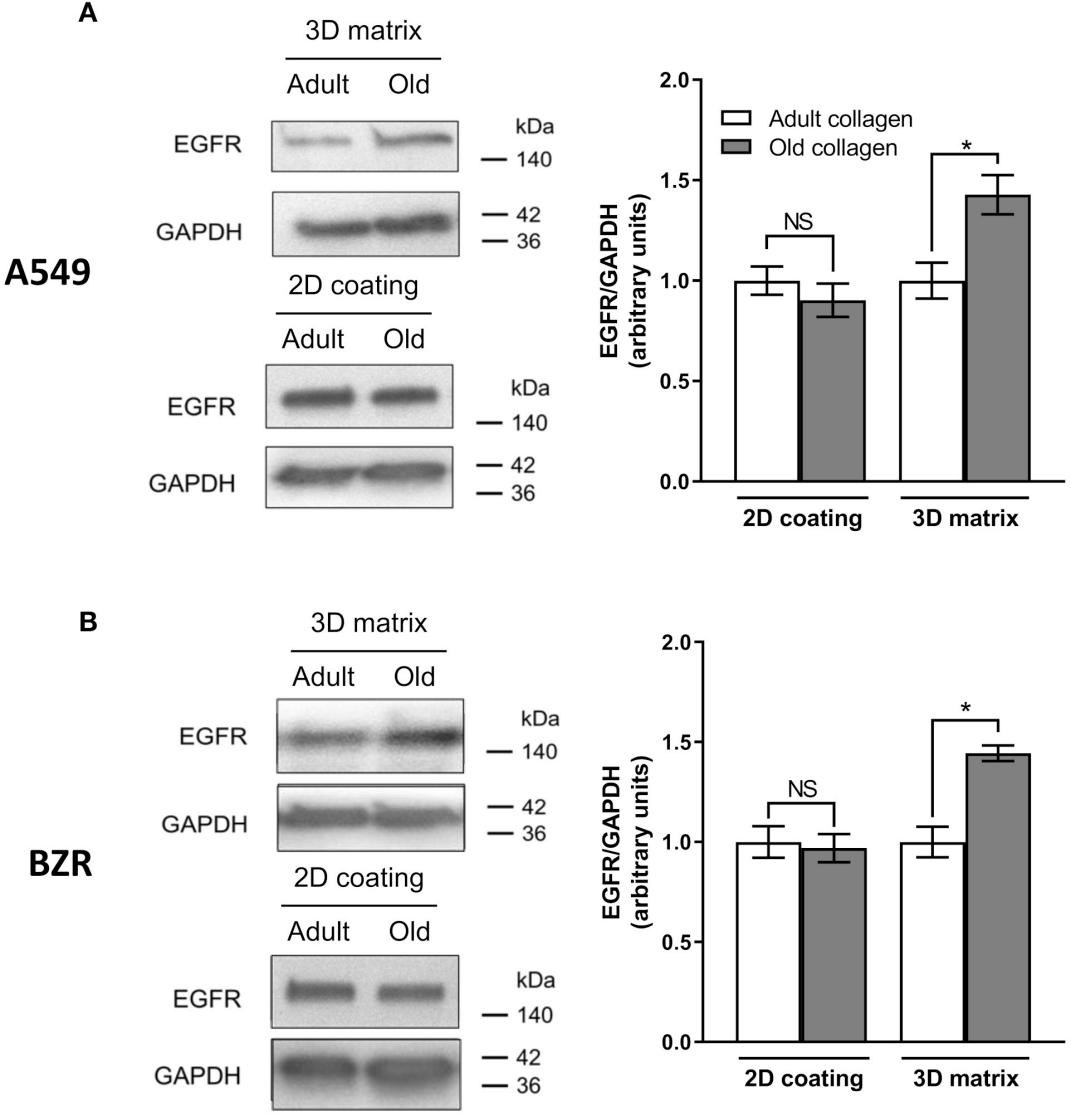
Effect of collagen aging on EGFR expression. **(A)** A549 cells and **(B)** BZR cells were cultured 96 h in adult and old 3D collagen matrices (upper panel) and adult and old 2D collagen coatings (lower panel). Western blot analysis was performed using anti-EGFR specific antibody. GAPDH was used as a loading control. The histograms show the ratio of EGFR expression relative to GAPDH. Values represent the mean ± SEM (*p < 0.05, N.S = Not significant).

The study reported by Chang et al. has shown that modification of collagen rigidity was able to increase EGFR expression level in A549 cells in a 2D model culture (8). Since changes occurring during collagen aging are also able to increase its rigidity (17, 18), we evaluated the effect of collagen aging on EGFR expression in A549 cells (Figure 4A) and in BZR cells (Figure 4B). While EGFR expression level was similar in both collagens in a 2D coating model, we show that in a 3D matrix model, EGFR expression level was increased by 1.5-fold for A549 and BZR, in old collagen when compared to the adult.

### Collagen Aging Promotes EGFR Phosphorylation

**Figure 5:**
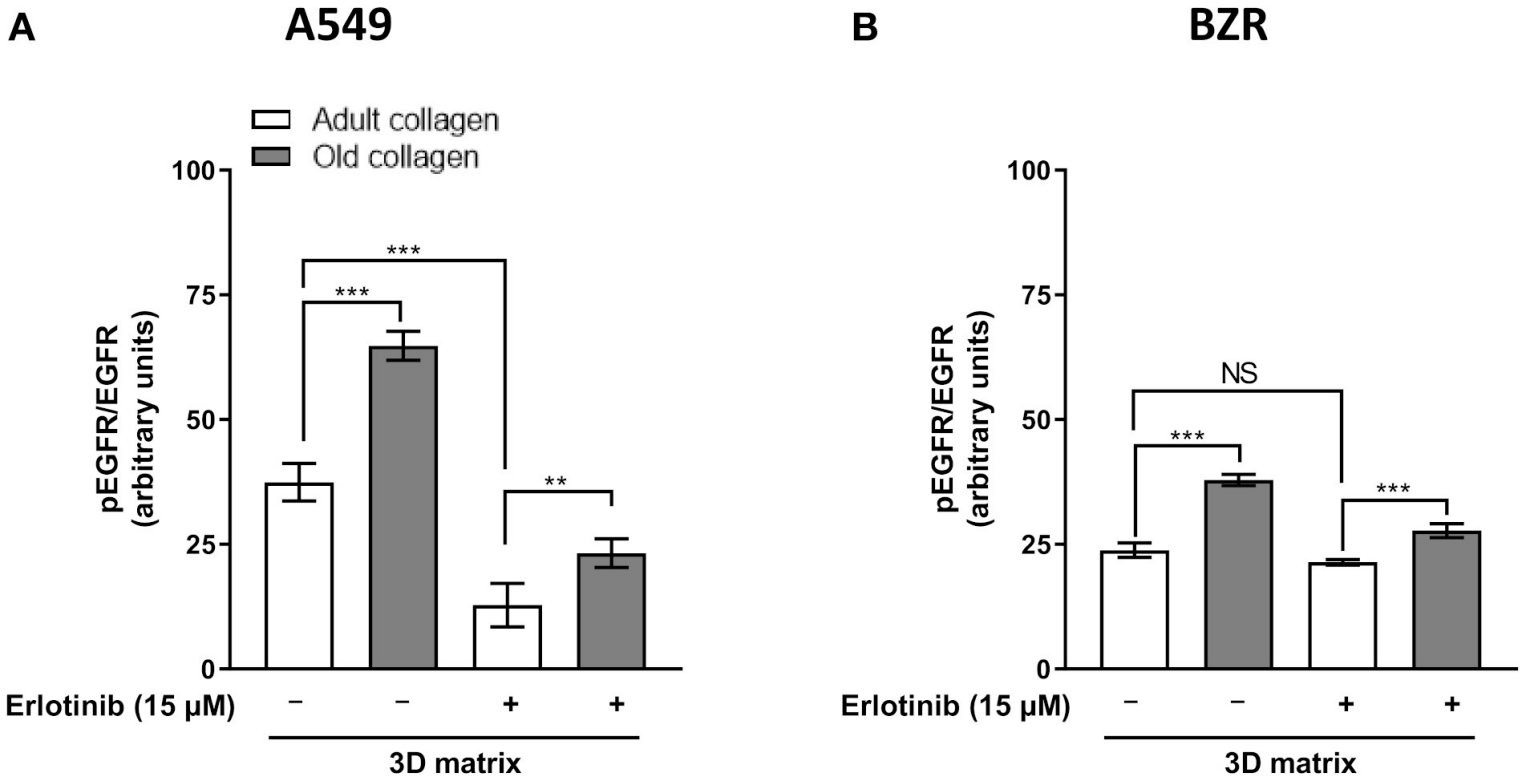
Effect of collagen aging on EGFR activation. **(A)** A549 cells and **(B)**. BZR cells were seeded in adult and old type I collagen 3D matrices at a density of 10 × 10^4^ cells/mL. After 24 h of culture, culture medium was replaced with medium containing erlotinib at a concentration of 15 μM or DMSO (vehicle), and 72 h later pEGFR/total EGFR ratio was assessed by flow cytometry. Values represent the mean ± SEM (**p < 0.01, ***p < 0.001).

EGFR phosphorylation level was analyzed in A549 and BZR cells cultured in 3D collagen matrix model. As shown in Figure 5A, EGFR phosphorylation level was 2-fold higher in old collagen than in adult collagen. However, in the presence of erlotinib, EGFR phosphorylation was significantly decreased in both collagens, but remains 2-fold higher in old collagen when compared to adult collagen. The same results were obtained for BZR cells (Figure 5B), with an EGFR phosphorylation level 1.5-fold higher in old collagen than in the adult one

## Discussion

In addition to EGFR amplification, NSCLC presenting EGFR mutations represent 10–15%. Exon 19 deletion and L858R mutations in exon 21 represent 85–90% of all mutations. Until recently, the best therapeutics for NSCLC presenting EGFR mutations were gefitinib, afatinib, and erlotinib (5). However, acquired resistance to those inhibitors are likely to occur within 10–12 months after the beginning of the treatment, mainly due to a second mutation T790M in exon 20. This mutation increases EGFR affinity for ATP and decreases treatment efficacy. New third generation inhibitors directed against this mutation have shown a better efficacy. It is the case for osimertinib, which was approved by FDA in 2016 (5). However, C797S mutation has been identified as a novel mechanism of resistance to osimertinib (21). Indeed, osimertinib has been described to bind to cysteine at position 797 of EGFR, which is replaced by serine after this secondary mutation.

Works from other groups have shown that tumor microenvironment was able to modulate sensitivity to targeted therapies and to induce drug resistance. This *de novo* resistance is called Environment-Mediated Drug Resistance (EMDR) and allow tumor cells to tolerate the effect of a therapy upon the first treatment (7). EMDR can be separated in two groups: EMDR due to soluble factors which is called Soluble Factor-Mediated Drug Resistance (SFM-DR), and EMDR due to adhesion process between the tumor cell receptors and extracellular matrix (ECM) proteins which is called Cell Adhesion-Mediated Drug Resistance (CAM-DR).

Type I collagen is the major constituent of ECM and can be found in several organs such as skin, breast, and lungs. Type I collagen plays an important role in tissues scaffolding but is also important in CAM-DR (7). In the context of lung carcinoma, Chang et al. have shown that artificial modification of type I collagen rigidity in a 2D coating model was able to increase the expression of EGFR in A549 cells, and to induce consequently resistance to erlotinib (8).

During chronological aging, type I collagen undergoes post-translational modifications such as glycation, that leads to an increase in AGE level (15), which is associated in turn to an increase in cross-links (16) and thus to modifications of collagen organization and rigidity (17, 18). The question we address here is whether the resistance to erlotinib observed by Chang et al., in collagen 2D model by modulating collagen properties artificially, could be observed in a 3D model that better mimics tumor organization *in vivo*, and in which collagen remodeling was induced by chronological aging (22). Our data shows that collagen aging increases EGFR expression in 3D collagen matrices. Interestingly, and at the opposite to the data obtained by Chang et al. we did not observe an increase of EGFR expression in the 2D model of collagen coating. In fact, if we compare the data obtained in 2D and 3D collagen models, the differential sensitivity of cells to erlotinib was observed only in the 3D collagen model. Moreover, the IC_50_ of erlotinib were generally higher in the 3D model when compared to 2D model. Such differential sensitivity could be explained by the fact that the 3D culture model is able to confer to epithelial carcinoma cells stem-like properties, which in turn decrease their sensitivity to therapies. In fact, cancer stem cell enrichment can induce a decrease in the sensitivity of glioblastoma to kinase inhibitors (23). This has also been reported for the tumorigenic potential in breast carcinoma (24). In fact, the authors suggested that 3D collagen matrix was able to generate breast carcinoma cells with stem-like properties.

The increase in EGFR expression observed with collagen aging in 3D was correlated to an increase in EGFR phosphorylation. These data are in agreement with those reported by Bertero and Lee groups. In fact, they have shown that ECM stiffening leads to an increase in YAP expression in cancers cells (25). According to this finding, another group has shown that the increase in YAP expression leads to EGFR TKI resistance in lung adenocarcinomas (26). In the present work, Erlotinib was able to inhibit EGFR activation in both adult and old collagens. However, due to the higher level in expression and phosphorylation of EGFR in old collagen before treatment, such levels remained significantly higher in old collagen when compared to adult collagen after treatment. This differential activation of EGFR after erlotinib treatment could explain the difference observed in terms of sensitivity to erlotinib in the two collagens.

Finally, according to the WHO, the global proportion of people over 60 is expected to increase from 12 to 22% by 2050, leading to changes in patterns of morbidity and causes of death. In addition, 30% cases of cancer are diagnosed in subjects aged 75 years and over of new cases. The importance of aging has been emphasized in the oncogenic mechanisms in the majority of tumors. Aging can also be one of the causes of therapeutic failure. However, its contribution to the therapeutic response is not sufficiently taken into account today and could partly explain the failure of many therapies in the elderly patient. Non-small-cell lung carcinoma deaths occur in patients that are 60 years old or older (27). Here we show that type I collagen modifications that occurs during aging protects lung cancer cells from erlotinib-induced cytotoxicity and apoptosis. This suggests that patient's age should be taken into account in the treatment of elderly patients.

## Data Availability Statement

The datasets generated for this study are available on request to the corresponding author.

## Ethics Statement

The animal procedure was approved by the local ethics committee in animal experimentation of Reims Champagne-Ardenne (C2EA, registration 56, France) and the experiments were performed in accordance with European directive 2010/63/UE.

## Author Contributions

HM and CS contributed to study conception and design. TS, LV, CC, CT, and CS performed experiments. TS, GC, CT, HM, and CS contributed to data analysis and interpretation. TS, GC, EB, HM, and CS contributed to manuscript writing and revision.

## Conflict of Interest

The authors declare that the research was conducted in the absence of any commercial or financial relationships that could be construed as a potential conflict of interest.

## References

[1] Barta JA Powell CA Wisnivesky JP. Global epidemiology of lung cancer. Ann Glob Health. (2019) 85:8. 10.5334/aogh.2419 30741509 PMC6724220

[2] Fitzmaurice C Dicker D Pain A Hamavid H Moradi-Lakeh M MacIntyre MF. The Global burden of cancer 2013. JAMA Oncol. (2015) 4:505–27. 10.1001/jamaoncol.2015.0735 26181261 PMC4500822

[3] Thun MJ Hannan LM Adams-Campbell LL Boffetta P Buring JE Feskanich D. Lung cancer occurrence in never-smokers: an analysis of 13 cohorts and 22 cancer registry studies. PLoS Med. (2008) 5:e185. 10.1371/journal.pmed.0050185 18788891 PMC2531137

[4] Travis WD Brambilla E Noguchi M Nicholson AG Geisinger K Yatabe Y International association for the study of lung cancer/american thoracic society/european respiratory society: international multidisciplinary classification of lung adenocarcinoma: executive summary. Proc Am Thorac Soc. (2011) 5:381–5. 10.1513/pats.201107-042ST 21926387

[5] Minari R Bordi P Tiseo M. Third-generation epidermal growth factor receptor-tyrosine kinase inhibitors in T790M-positive non-small cell lung cancer: review on emerged mechanisms of resistance. Transl Lung Cancer Res. (2016) 5:695–608. 10.21037/tlcr.2016.12.02 28149764 PMC5233880

[6] Pickup MW Mouw JK Weaver VM. The extracellular matrix modulates the hallmarks of cancer. EMBO Rep. (2014) 15:1243–53. 10.15252/embr.201439246 25381661 PMC4264927

[7] Meads MB Gatenby RA Dalton WS Environment-mediated drug resistance: a major contributor to minimal residual disease. Nat Rev Cancer. (2009) 9:665–74. 10.1038/nrc2714 19693095

[8] Chang CC Hsieh TL Tiong TY Hsiao CH Tung-Qian Ji A Hsu WT. Regulation of metastatic ability and drug resistance in pulmonary adenocarcinoma by matrix rigidity via activating c-Met and EGFR. Biomaterials. (2015) 60: 141–50. 10.1016/j.biomaterials.2015.04.058 26000960

[9] Henriet P Zhong ZD Brooks PC Weinberg KI DeClerck YA. Contact with fibrillar collagen inhibits melanoma cell proliferation by up-regulating p27KIP1. Proc Natl Acad Sci USA. (2000) 18:10026–31. 10.1073/pnas.170290997 10944199 PMC27660

[10] Assent D Bourgot I Hennuy B Geurts P Noël A Foidart JM. A membrane-type-1 matrix metalloproteinase (MT1-MMP)-discoidin domain receptor 1 axis regulates collagen-induced apoptosis in breast cancer cells. PLoS ONE. (2015) 10:e0116006. 10.1371/journal.pone.0116006 25774665 PMC4638154

[11] Saby C Collin G Sinane M Buache E Van Gulick L Saltel F. DDR1 and MT1-MMP expression levels are determinant for triggering BIK-mediated apoptosis by 3D type I collagen matrix in invasive basal-like breast carcinoma cells. Front Pharmacol. (2019) 10:462. 10.3389/fphar.2019.00462 31130862 PMC6509437

[12] Bartling B Desole M Rohrbach S Silber RE Simm A. Age-associated changes of extracellular matrix collagen impair lung cancer cell migration. FASEB J. (2009) 5:1510–20. 10.1096/fj.08-122648 19109409

[13] Saby C Buache E Brassart-Pasco S El Btaouri H Courageot MP Van Gulick L. Type I collagen aging impairs discoidin domain receptor 2-mediated tumor cell growth suppression. Oncotarget. (2016) 7:24908–27. 10.18632/oncotarget.8795 27121132 PMC5041879

[14] Saby C Rammal H Magnien K Buache E Brassart-Pasco S Van-Gulick L. Age-related modifications of type I collagen impair DDR1-induced apoptosis in non-invasive breast carcinoma cells. Cell Adhes Migr. (2018) 12:335–47. 10.1080/19336918.2018.1472182 29733741 PMC6363044

[15] Simm A Müller B Nass N Hofmann B Bushnaq H Silber RE. Protein glycation - Between tissue aging and protection. Exp Gerontol. (2015) 68:71–5. 10.1016/j.exger.2014.12.013 25536383

[16] Monnier VM Mustata GT Biemel KL Reihl O Lederer MO Zhenyu D. Cross-linking of the extracellular matrix by the maillard reaction in aging and diabetes: an update on “a puzzle nearing resolution.” Ann N Y Acad Sci. (2005) 1043:533–44. 10.1196/annals.1333.061 16037276

[17] Aït-Belkacem D Guilbert M Roche M Duboisset J Ferrand P Sockalingum G. Microscopic structural study of collagen aging in isolated fibrils using polarized second harmonic generation. J Biomed Opt. (2012) 17:080506–1. 10.1117/1.JBO.17.8.080506 23224157

[18] Van Gulick L Saby C Morjani H Beljebbar A. Age-related changes in molecular organization of type I collagen in tendon as probed by polarized SHG and raman microspectroscopy. Sci Rep. (2019) 1:7280. 10.1038/s41598-019-43636-2 31086263 PMC6513820

[19] Garnotel R Rittie L Poitevin S Monboisse JC Nguyen P Potron G. Human blood monocytes interact with type I collagen through alpha x beta 2 integrin (CD11c-CD18, gp150-95). J Immunol. (2000) 164:5928–34. 10.4049/jimmunol.164.11.5928 10820275

[20] Shan F Shao Z Jiang S Cheng Z. Erlotinib induces the human non-small-cell lung cancer cells apoptosis via activating ROS-dependent JNK pathways. Cancer Med. (2016) 11:3166–75. 10.1002/cam4.881 27726288 PMC5119972

[21] Niederst MJ Hu H Mulvey HE Lockerman EL Garcia AR Piotrowska Z. The allelic context of the C797S mutation acquired upon treatment with third-generation EGFR inhibitors impacts sensitivity to subsequent treatment strategies. Clin Cancer Res. (2015) 17:3924–33. 10.1158/1078-0432.CCR-15-0560 25964297 PMC4587765

[22] Edmondson R Broglie JJ Adcock AF Yang L. Three-dimensional cell culture systems and their applications in drug discovery and cell-based biosensors. Assay Drug Dev Technol. (2014) 4:207–18. 10.1089/adt.2014.573 24831787 PMC4026212

[23] Fernandez-Fuente G Mollinedo P Grande L Vazquez-Barquero A Fernandez-Luna JL. Culture dimensionality influences the resistance of glioblastoma stem-like cells to multikinase inhibitors. Mol Cancer Ther. (2014) 13:1664–72. 10.1158/1535-7163.MCT-13-0854 24723451

[24] Chen L Xiao Z Meng Y Zhao Y Han J Su G. The enhancement of cancer stem cell properties of MCF-7 cells in 3D collagen scaffolds for modeling of cancer and anti-cancer drugs. Biomaterials. (2012) 33:1437–44. 10.1016/j.biomaterials.2011.10.056 22078807

[25] Bertero T Oldham WM Grasset EM Bourget I Boulter E Pisano S. Tumor-stroma mechanics coordinate amino acid availability to sustain tumor growth and malignancy. Cell Metab. (2019) 1:124–40.e10. 10.1016/j.cmet.2018.09.012 30293773 PMC6432652

[26] Lee TF Tseng YC Nguyen PA Li YC Ho CC Wu CW. Enhanced YAP expression leads to EGFR TKI resistance in lung adenocarcinomas. Sci Rep. (2018) 1:271. 10.1038/s41598-017-18527-z 29321482 PMC5762715

[27] Ferlay J Ervik M Lam F Colombet M Mery L Piñeros M Global Cancer Observatory: Cancer Today. (2018). Available online at: https://gco.iarc.fr/today (accessed February 20, 2020).

